# Improved Hybrid Model for Obstacle Detection and Avoidance in Robot Operating System Framework (Rapidly Exploring Random Tree and Dynamic Windows Approach)

**DOI:** 10.3390/s24072262

**Published:** 2024-04-02

**Authors:** Ndidiamaka Adiuku, Nicolas P. Avdelidis, Gilbert Tang, Angelos Plastropoulos

**Affiliations:** 1 Integrated Vehicle Health Management Centre (IVHM), School of Aerospace, Transport and Manufacturing, Cranfield University, Bedfordshire MK43 0AL, UK; 2 Centre for Robotics and Assembly, School of Aerospace, Transport and Manufacturing (SATM), Cranfield University, Bedfordshire MK43 0AL, UK

**Keywords:** autonomous navigation, object detection, obstacle avoidance, mobile robot, deep learning, vision

## Abstract

The integration of machine learning and robotics brings promising potential to tackle the application challenges of mobile robot navigation in industries. The real-world environment is highly dynamic and unpredictable, with increasing necessities for efficiency and safety. This demands a multi-faceted approach that combines advanced sensing, robust obstacle detection, and avoidance mechanisms for an effective robot navigation experience. While hybrid methods with default robot operating system (ROS) navigation stack have demonstrated significant results, their performance in real time and highly dynamic environments remains a challenge. These environments are characterized by continuously changing conditions, which can impact the precision of obstacle detection systems and efficient avoidance control decision-making processes. In response to these challenges, this paper presents a novel solution that combines a rapidly exploring random tree (RRT)-integrated ROS navigation stack and a pre-trained YOLOv7 object detection model to enhance the capability of the developed work on the NAV-YOLO system. The proposed approach leveraged the high accuracy of YOLOv7 obstacle detection and the efficient path-planning capabilities of RRT and dynamic windows approach (DWA) to improve the navigation performance of mobile robots in real-world complex and dynamically changing settings. Extensive simulation and real-world robot platform experiments were conducted to evaluate the efficiency of the proposed solution. The result demonstrated a high-level obstacle avoidance capability, ensuring the safety and efficiency of mobile robot navigation operations in aviation environments.

## 1. Introduction

The aviation sector is increasingly integrating mobile robot solutions into its operational framework for activities, including inspection and maintenance tasks. The robot systems are designed to perform diverse tasks, including inspecting aircraft components for defect identification, collecting data, and transporting tools and equipment. However, safely navigating in a complex aviation environment like the smart hangars is difficult for these robots [1]. This necessitates the development of enhanced navigation algorithms with sensor technologies crucial for the robot to navigate safely and effectively in such workspace. Recent developments in vision-based navigation systems have seen significant progress, leveraging robot operating system (ROS) navigation stack local and global planners [2]. ROS 1 provides a modular and flexible set of software packages for developing autonomous navigation applications. The ROS navigation stack planners work in conjunction to generate obstacle-free routes from the robot’s current position to its goal destination. The local planner includes the time elastic band (TED) [3] and dynamic windows approach (DWA) [4] algorithm, while the global planners consist of Dijkstra and A*, employed as default components of the ROS navigation stack. The Dijkstra algorithm is known among global planners for its efficacy in finding the shortest path by exploring the entire space [5]. This can be computationally expensive, time-consuming, and impractical in dynamic environments, making it more suitable in static environments with predefined obstacles. The increasingly complex and real-world demands in dynamic environments limit the practical application of this algorithm. Looking at the smart hangar environment where the environment is dynamic and requires real-time responsiveness, the changing conditions of environmental factors necessitate navigation solutions that are adaptive and capable of real-time decision-making [6]. Rapidly exploring random tree (RRT) planner is among the global planners that employ sampling strategy, sampling points by exploring the robots’ configuration space and incrementally building a tree structure based on these samples. This enables the robot to explore diverse areas where the obstacle may appear or move and adapt its path in response to changing conditions. While it is capable of adapting to changes in the environment, it may not react quickly to dynamic obstacles and rapidly changing conditions. Consequently, there is a need for more advanced hybrid approaches that can effectively handle the unpredictable nature of real-world hangar environments, ensuring safe, efficient, and real-time navigation of mobile robots [7]. The integration of advanced learning-based solutions, which can learn, process, and adapt to varying environmental conditions, has shown significant improvement for an efficient mobile robot solution in complex settings. Some of these solutions have combined the strength of machine learning [8,9], sensor fusion [10], or other conventional navigation techniques [11,12] to create robust systems capable of real-time decision-making and adaptation for robot navigation. Machine learning techniques, such as YOLO models, play a significant role in object detection and real-time capabilities [13], enabling the robot to detect varying obstacles in its environment as it navigates. While YOLO does not directly drive the robot’s path planning and obstacle avoidance approach, it enhances the robot’s situational awareness by continuously identifying obstacles in the scene. This information can be utilized by the robot to make more informed decisions and update its RRT-based path to navigate around obstacles in real time.

This paper proposes an improved hybrid framework that integrates the capability of the RRT-based ROS navigation stack with the YOLOv7 object detection model, which harnesses sensor information from LiDAR and camera systems to enhance the adaptability and navigation functionalities of mobile robots in complex and changing environments. This brings improvement to an established NAV-YOLO framework in the ongoing research project that leverages path planning proficiency of RRT and sensor fusion to significantly improve the precision and reliability of autonomous robot navigation in environments characterized by unpredictability and variable obstacles. The camera sensor, while providing high-resolution visual information, is constrained by a limited field of view, and this is complimented by integrating LiDAR input that provides 360-degree environmental coverage. This broadens the scope of obstacle detection and significantly enhances the mobile robot’s ability to perceive, navigate, and interact with a wide range of environmental features. The object data detected by the pre-trained YOLO node [14] from the camera stream is integrated with laser scan data from the LiDAR sensor within the cost map file of the ROS ecosystem. This communicates multi-dimensional environmental information to RRT and DWA planners within the navigation system and facilitates the robot’s accurate obstacle avoidance decision-making and path-planning capabilities in real time. This framework is simulated and validated in turtlebot3-enabled ROS-Gazebo environment and physical robot. The experiment showed an intelligent obstacle detection and avoidance solution that significantly enhanced robot navigation capabilities for aircraft inspection in a hangar environment. The general contribution includes,

1. RGB camera and LiDAR sensor fusion to broaden the robot’s field of view in complex and dynamic settings.
2. Integration of customized pre-trained YOLOv7 model into ROS framework for real-time capabilities and accurate detection of varying and unstructured environmental obstacles.
3. Enhancement of NAV-YOLO with RRT global planner for effective real-time obstacle avoidance and path planning.
4. Comparative analysis of ROS navigation stack, NAV-YOLO, and proposed RRT enables NAV-YOLO to validate improved performance.

The rest of the paper is structured in four parts: Section 1 discusses related works that have contributed to obstacle detection with YOLO, sensor fusion capability, and robot navigation with the ROS framework. Section 2 details the methodology, describing the YOLOv7 version of the object detection model, the structural component of the RRT path planning algorithm, and an overview of the development process. In Section 3, the experimental analysis was performed, analyzing the detection and path planning capability of the proposed model related to NAV-YOLO and the baseline model. Finally, Section 4 presents the summary and future direction.

## 2. Related Work

Diverse approaches have been employed in expanding the potential application of robotics, especially around safe navigation in complex environments. The combination of traditional algorithms and cutting-edge machine learning techniques has shown significant efficiency and reliability in obstacle detection and avoidance for a safe robot navigation experience. However, adaptability in environments densely populated with varying obstacles and the need for an efficient real-time response for optimal obstacle avoidance and path planning have become major challenges in many applications. The study presented in [11,12] proposed a CNN-based neural RRT model, similar to research work in [15] that utilized conditional variational autoencoder (CVAE) to enhance the path planning process using a nonuniform sampling distribution method, but environmental obstacles information was not given keen consideration in these methods. Canglong et al. [16] applied a combined solution based on ROS and CNN-based end-to-end learning that utilized datasets acquired from a low-level camera. This was applied in a structured environment and will struggle in real-time complex scenarios.

Another work in [17] uses object detection based on a vision system and deep learning to detect obstacles and accelerate path planning. However, obstacle avoidance capability is limited. This was addressed in [18] by developing a solution that learns obstacle representation in complex environments using LiDAR sensors to demonstrate generalization in unknown features and enhance path planning ability. A sensor-based learning system was presented in [19] that uses an integrated sampling-based planner with deep learning architecture to improve obstacle avoidance and path planning. Here, LiDAR’s inability to detect rich environmental information affects the accuracy of obstacle detection and avoidance. From another view, the author in [20] employed depth camera information to develop the YOLOv4 object detection model and enhanced the path planning process with RRT while integrating sensor fusion information with deep reinforcement learning. The work in [21] also applied multimodal LiDAR and camera information for the object detection process using the pre-trained CNN technique to improve autonomous vehicle perception.

This paper proposed a new navigation methodology that learns from perceived environmental features for precise obstacle avoidance and path selection decision-making, and this facilitates real-time adaptability and responses in complex scenes. Our proposed integration framework of YOLOv7 and ROS framework techniques presents a significant advancement with sensor fusion in robot navigation capabilities, enabling safe and efficient operation in complex and unpredictable settings and opening new avenues for autonomous robot deployment in diverse industries.

## 3. Methodology

This project presents the development of a vision-based hybrid model for obstacle avoidance and path planning in mobile robotics, innovatively merging conventional ROS navigation methods with advanced YOLO deep learning techniques. The approach addresses safety needs critical to autonomous robot navigation, capable of detecting a wide range of obstacles. Autonomous robot navigation not only demands high-precision obstacle detection capability but also needs to respond in real time to complexities and change MRO scenes to ensure that the robot can make timely and accurate decisions. The navigation system is centered on integrating RRT with DWA planners to combine the efficiency of the RRT in optimal path planning and DWA in real-time obstacle avoidance. This solution is enhanced by integrating LiDAR/Camera sensor fusion and the YOLO object detection model, which allows mobile robots to navigate around obstacles with precision while optimizing paths in real time.

### 3.1. CNN-Based YOLOv7 Model

YOLO is one of the many CNN-based computer vision models that is based on a single-stage, end-to-end training and detection process [13]. With the capability to perform multiple detections simultaneously, its improved variants, like YOLOv7, noted with superior accuracy and speed of inference, outperform other related object detection models like SSD [22] and Faster RCNN [23] two-stage object detection models. While these models have been instrumental in object detection, they often lag in speed and optimization efficiency, especially in complex environments with varied obstacle structures. Over time, a series of successive iterations of the YOLO framework have been conceived and are presently undergoing further development [24]. YOLOv7 is among the latest versions in the YOLO series of real-time object detection models, built for real-world challenges, and was officially released in 2022 [25]. The concept involves data processing, including image resizing, data augmentation, and the use of a convolution neural network (CNN) as the backbone architecture for feature extraction. Also, it uses anchor boxes to predict bounding boxes for objects, ensuring that anchor boxes closely match object sizes and shapes in the data. A recent study [26] compared YOLO variants, including YOLOv5, as faster and more lightweight than the previous YOLO, while YOLOv7 showed improvements in speed and accuracy, which are relevant in this manuscript for real-time robot navigation.

Our previous experiment with YOLOv5 showed reasonable speed and accuracy in the simulation environment but a limited success rate when deployed on the physical turtlebot3 robot platform obviously due to limited hardware capacity. This necessitated the experimentation with an improved YOLO variant for faster speed in frames per second (FPS) and enhanced accuracy. One of the metrics used to evaluate the model’s performance as explained in our previous work in [14] includes mean average precision (mAP), which is the mean of the average precision value calculated for each object class. A higher mAP indicates good performance in object detection. The intersection over union (IoU) is another metric that measures the overlap between the predicted bounding box and the ground truth bounding box. A higher IoU indicates better accuracy in detected objects. The FPS measures the speed at which the model can process images and higher FPS indicates faster processing and model efficiency.

### 3.2. ROS Planners

ROS planners are part of the ROS navigation stack algorithms that provide path planning and navigation functionalities in the ROS framework. The ROS framework is a collection of libraries and conventions used in developing robot solutions [27]. The default ROS path planning algorithms include Dijkstra and DWA global and local planners [28]. Other advanced path-planning algorithms have been developed over the years, like the RRT, which, although not a native part of the ROS navigation stack, can be integrated to optimize path-planning functions. RRT is an incremental sampling-based algorithm [29] with a tree structure that selects a random state xrand from a uniform distribution in every iteration and then identifies the nearest vertex xnearest in the tree state [30]. Every successful connection establishes a new state, xnew, ensuring a feasible transition where new vertexes and edges are formed between xnearest and xnew. This iteratively expands the tree structure, exploring the state space and balancing the computational demand until target conditions are met. To address some issues encountered during the experiments, specific parameters were updated within the RRT ‘move_base’ package. The goal tolerance was changed from the default 0.5 to 1 m to reduce computational issues that made most of the integrated package struggle to function and improve the robot’s ability to smoothly align with the target location. The number of iterations was also reduced from 4000 to 1000 to make the path search faster. The branch length was realized to be short and affected the robot’s ability to align with the generated path. This was increased from 0.2 to 0.6 to address this problem. The RRT allows exploring the wide space of the hangar. For this study, the RRT is integrated to enhance the functionalities of the dynamic windows approach (DWA) local planner.

The DWA local planner is specifically designed for local trajectory planning and obstacle avoidance. It takes into account the robot’s current velocity, acceleration limits, and the immediate environment to calculate the safe and feasible path toward the goal. DWA operates by creating a dynamic window of possible commands (linear and angular velocities) that the robots can execute, considering their dynamics and the feasibility of the trajectory [4]. The planner utilizes the local costmap, which represents the environment around the robots and the detected obstacles, to evaluate the potential collision of simulated trajectories [27]. DWA is highly configurable with parameters that can be fine-tuned for specific navigation tasks, such as velocity ranges, acceleration limits, etc. While DWA is adept at avoiding obstacles in less complex environments [31], its performance in highly cluttered or constrained environments, like the MRO hangars, may be suboptimal. In such environments, DWA benefits from being paired with complimentary algorithms to achieve optimal performance, as it may require additional strategies to navigate effectively through dense and unpredictable spaces.

### 3.3. Sensor Fusion

One major research interest in intelligent robotics is the integration of multisensory capabilities to expand and enhance a robot’s perception of its environment and navigation operations. This provides a more comprehensive understanding of the surrounding robot’s environment and enables it to make more accurate and informed decisions, adapting to varying environmental structures and conditions compared to relying on an individual sensor [32]. This work integrates LiDAR and camera sensor devices; LiDAR provides geometry of the surroundings and a 360-degree field of view [33], while transformed camera frames from YOLO detectors add rich definitions to environment variables. The data from LiDAR and YOLO-detected camera frames are registered in the robot’s costmap. The costmap is a 2D grid-based representation of the environment around the robots where the fused data stream continuously updates the costmap, providing the planners with a dynamic and accurate representation of the environment [34]. This information is then used in conjunction with the planners to enhance the robot’s perception capabilities and make better-informed decisions for obstacle detection, avoidance, and path planning.

The LiDAR data are published using sensor_msg/LaserScan, while YOLO-detected object information is integrated with sensor_msg/Image ROS package. The coordinates of the detected objects are transformed to be in the same reference as the robot’s navigation system. This information is updated in the ROS navigation stack costmap_common_params.yaml file, where the obstacle layer that represents obstacles detected by lidar is configured in local_costmap_params.yaml, and the YOLO-detected object layer is created as an extension in the costmap for YOLO information. The obstacle layers from LiDAR and YOLO layers are included in the move_base_params.yaml file. The combined information improves the robot’s field of view in the environment. This integration strives to capitalize on the strengths of both sensor types [32,35], overcoming the limitations posed by LiDAR’s temporal resolution while leveraging its wide field of view for robust obstacle detection and avoidance.

### 3.4. System Architecture

**Figure 1:**
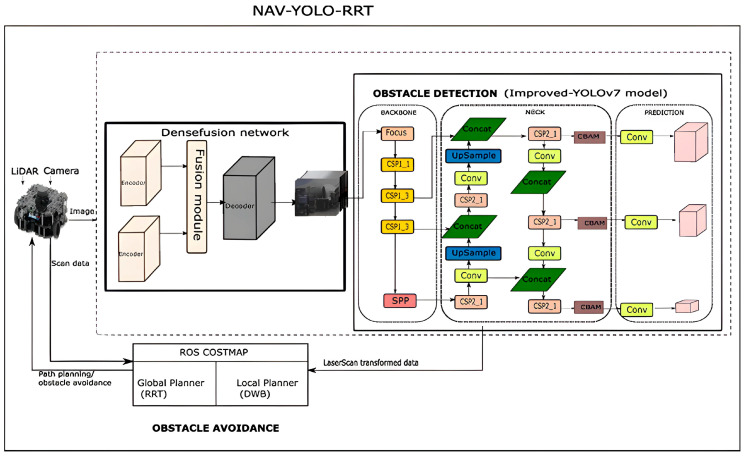
NAV-YOLO-RRT system architecture.

The solution was first established by combining pre-trained YOLOv5 [14] and the default ROS navigation stack to develop a system called NAV-YOLO that showed a need for improvement after evaluation [36]. The NAV-YOLO approach interfaced with YOLO and default ROS planners, which include Dijkstra and DWA planners. The YOLO identifies and localizes the object in the robot environment based on the camera feed and publishes the detected object’s information using bounding boxes and class labels on a ROS topic. This is configured in the costmap, which acts as a bridge between YOLO and the ROS planners. The ROS planners then utilize the updated costmap, which represents the interface between the robot’s environment and ROS planners, to generate safe and efficient navigation paths. The proposed solution in this manuscript followed the same approach but incorporated pre-trained YOLOv7, in tune with our previous work [14], and the RRT algorithm to enhance the performance of the NAV-YOLO. This development is structured in two modules, the obstacle detection and obstacle avoidance systems, as shown in Figure 1.

#### 3.4.1. Obstacle Detection Module

This system focuses on the accurate perception of environmental features using camera sensors to generate custom datasets for YOLOv7 accurate object detection. The YOLOv7 tiny variant was developed and pre-trained using successful techniques developed in our previous work [14]. From the previous work perspective, the contribution is to enhance the object detection model using the improved YOLO model. This approach includes capturing a wide range of obstacles found in MRO hangars, including tools, vehicles, and structural elements in the real world and in simulation, and using these datasets to train the model. LLVIP [35] public datasets were also integrated to expand the capability of recognizing objects in diverse scenarios considering low-light conditions, which are common in certain areas of the MRO hangar or during specific times of the day. For data fusion, the DenseFuse network was first used to fuse RGB and thermal images from real-world and LLVIP datasets to expand the model’s capability to accurately detect obstacles in low-light conditions. The architecture of the YOLO model was slightly adjusted to make it suitable for real-time application. This involves the integration of a convolution block attention mechanism module (CBAM), which improves performance by attention focusing on relevant features and suppressing background noise to prioritize important object details relevant for real-time detection [37]. The datasets are merged and pre-processed to suit YOLOv7 input requirements. The pre-processing process includes data annotation, data augmentation with flipping and cropping techniques, and image resizing to 640 × 640 dimensions, which were performed using the Roboflow annotation tool [38]. The training process was fine-tuned using a default hyperparameter, batch size of 20, and 640 × 640 resolution through iterations. The YOLOv7 was trained offline and evaluated to attend an accuracy level of about 68–70%, which indicates that the model can identify obstacles accurately within the input image even in low light conditions. This also ensures that the model maintains the required performance capable of running on the robot’s onboard hardware with limited computer resources. The model is trained and validated to ensure that the model’s inference is fast and reliable to allow real-time processing in ROS. The customized YOLOv7 model and weights file were saved and transferred to a dedicated ROS package with custom nodes that load the model and weights.

The YOLO node subscribes to the image topic to perform object detection on the received image. The detected result is published and transformed into a laser scan format for more informed navigation decisions. This is configured in the ROS navigation costmap, which interfaces with the ROS planners. The paper leverages YOLOv7 object detection capabilities to enhance the robot’s ability to better perceive its environment, enabling it to make more informed decision-making in navigation tasks. The RRT can generate feasible paths from the robot’s current position to the goal based on YOLO’s object detection results (detected objects) and can dynamically update the environment map, avoiding collisions with detected obstacles during path planning. YOLO also provides real-time updates on the positions and trajectories of dynamic obstacles, allowing DWA to adjust the robot’s velocity and steering commands accordingly to avoid collisions and ensure safe and efficient navigation.

#### 3.4.2. Obstacle Avoidance Module

To implement the obstacle detection module in the ROS ecosystem, YOLO detection and depth data nodes are created to load a pre-trained YOLO model. The camera image stream is subscribed to and processed to detect obstacles published with bounding box information. The bounding box coordinates with the classification type and confidence scores for each detected object are generated. With the depth map and camera properly calibrated and aligned to the same resolution and dimensions, the bounding box center is mapped to the depth image to extract the depth value at the center. This typically is the value of the distance from the sensor to the object at that point. The center of the bounding box can be calculated using the equation below.

bbox = x, y, w, h (from YOLO detection)(1)

(2) $$cx=x+\frac{w}{2}$$

(3) $$cy=y+\frac{h}{2}$$

where bbox is the bounding box defined by its top-left corner (x, y) and its dimensions (width w and height h). And cx and cy represent the center coordinates.

However, the process of deriving laser scan data from the center coordinates assumes that the detected object is solid, without any gaps, as is the case with standing humans or chairs. This was addressed by generating laser scan information from the entire width of the image at the center of the object’s height instead of at the center of the camera. This adaptation will allow for a more comprehensive representation of the object’s spatial extent, accommodating scenarios where there might be gaps or spaces within the object’s outline. This adjustment will enhance the fidelity of the generated laser information, contributing to more accurate and reliable obstacle detection and avoidance, especially in situations involving objects with varying shapes and configurations, and will be expanded in our next project phase.

#### 3.4.3. Converting Pixel Coordinates to Camera Coordinates

The key parameters for this transformation include the 2D coordinate image from the YOLO detector and camera intrinsic matrix with key parameters, including focal length (fx, fy) and principal point (cx, cy) of the camera shown in the Equations (1) and (2). The 2D coordinates from the image frame (u, v) are transformed to 3D coordinates in the camera’s coordinate (X, Y, Z) relative to the camera’s position and orientation to understand the objects’ spatial positioning in the real world from their positions in the image. This is shown in the Equations (1) and (2).

(4) $$X=\left(x\_obj-cx\right)\times \frac{z\_obj}{fx}$$

(5) $$Y=\left(y\_obj-cy\right)\times \frac{z\_obj}{fy}$$

(6) Z=z_obj=0

where x_obj and y_obj represents detected object coordinates, and z_obj represents depth estimation (assuming ground plane projection Z = 0). After obtaining the 3D points in the camera coordinates system, the points are transformed to the robot’s coordinate using extrinsic parameters (rotational matrix R and translation vector t) that describe the camera’s orientation and position relative to the robot’s coordinate. The equation is shown below as:

(7) $$\left[\begin{array}{c} X\\ Y\\ Z \end{array}\right]=R*\left[\begin{array}{c} x\_obj\\ y\_obj\\ z\_obj \end{array}\right]+t$$

with the robot’s center coordinate transformed in laser scan frame, considering its geometry and current pose, the Euclidean distance from each detected object to the robot’s center can be calculated [39]. That is the distance between two points in the space and is computed using the below formula.

(8) $$Distance=\sqrt{{\left(x\_obj–X\right)}^{2}+{{\left(y\_obj–Y\right)}^{2}+\left(z\_obj–Z\right)}^{2}}$$

**Figure 2:**
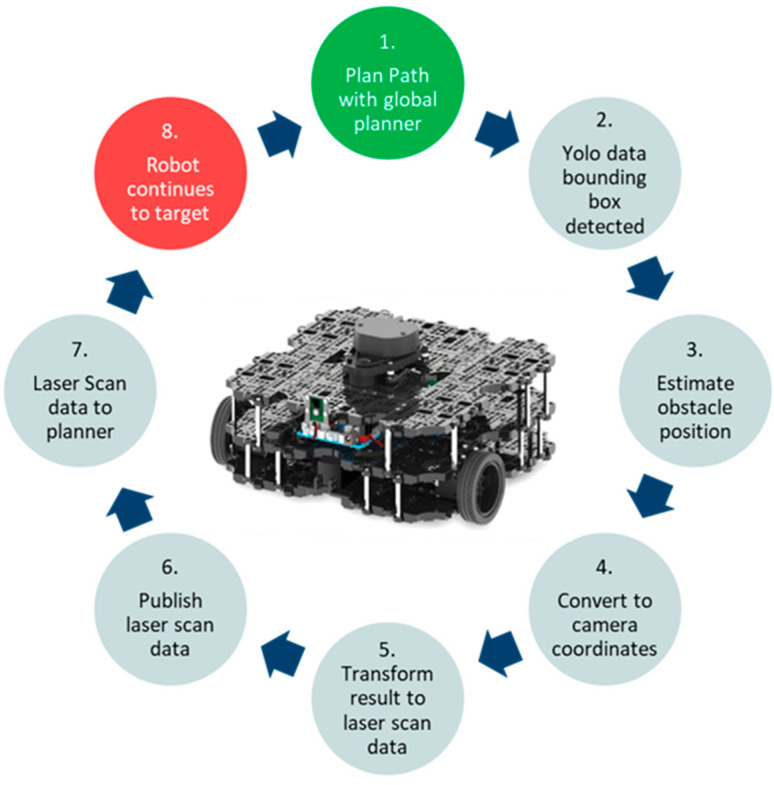
Detected objects transformation workflow.

This distance is employed to identify potential collisions and plan safe paths around the obstacles. The overall process of detection to transformation is represented in the workflow shown in Figure 2.

#### 3.4.4. Data Fusion

Having YOLO detection transformed, the detected objects are projected onto the LiDAR laser scan by associating it with the corresponding position in the LiDAR scan. The LiDAR sensor provides 360-degree scans of the environment, and each scan point represents distance and angle information relative to the robot’s position. The system utilizes sensor_mgs/LaserScan ROS packages to subscribe to LiDAR data, and the coordinates are transformed with the ROS *tf* package using the robot base frame as the reference. This ensures that camera images and LiDAR scans are represented in the same coordinate system. The transformed LiDAR data and YOLO-detected object nodes are configured in the costmap, with ROS observation source layers configured to subscribe to scan_1 and scan_2, which represent the LiDAR scan as/*trans_scan* and YOLO-detected object node as/*scan_yolo*, respectively. The transformed data are fused together and published as an integrated representation of the environment.

#### 3.4.5. Costmap Configuration

**Figure 3:**
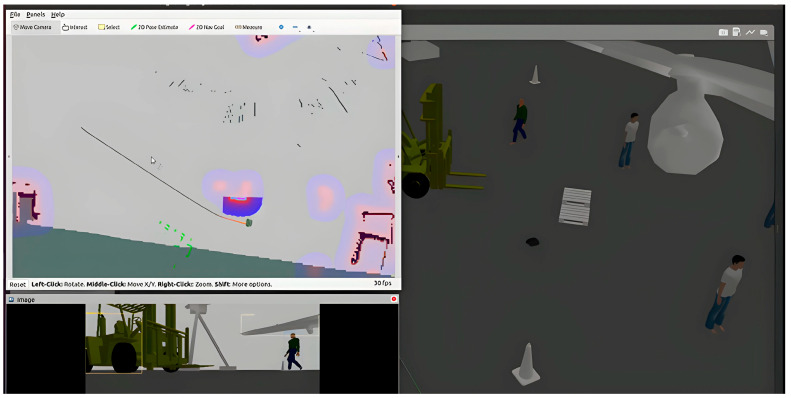
Robot navigation showing ROS path planning and YOLO object detection.

The resulting laser scan data are configured in the costmap to update costmap cells [27]. The cotsmap is a grid-based representation of the robot’s environment where each cell has a cost associated with traversing through it. The cells that correspond with the position of obstacles are assigned higher costs, while free cells are given lower costs [40]. Also, the LiDAR data are integrated into the *costmap_common_params.yaml* file to specifically expand the robot’s field of view. The costmap dynamic layer parameters, like the obstacle_range, update frequency, and resolution, are set to continuously update and accurately reflect the current environment. The ROS navigation stack path planners, including RRT and DWA, access the costmap to generate collision-free paths, where it considers the cost values of cells to avoid high-cost areas representing obstacles and determine traversable routes. The RVIZ visualization tool was employed to observe updates with necessary adjustments to enhance path planning algorithm control decisions in planning a safe route around the obstacle while navigating to the target location. Figure 3 gives an overview of the navigation work of the integrated ROS path planner and YOLO object detection solution. While the robot is navigating through its environment, Figure 3 shows the replanning capability as well as the object detected as it navigates around obstacles.

## 4. Experiment

**Figure 4:**
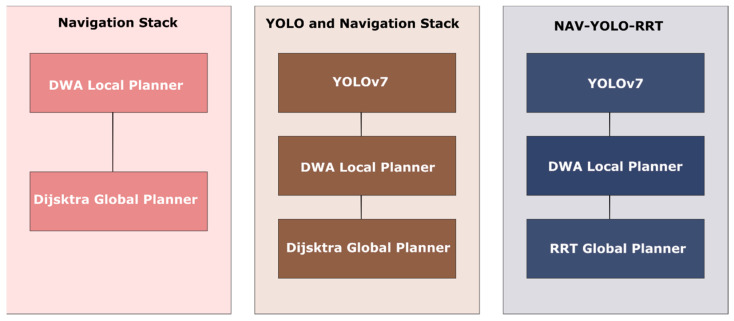
Comparison of NAV-YOLO methods.

The improved NAV-YOLO model was developed and evaluated in the ROS_Gazebo simulation environment. Gazebo is a robot simulation framework integrated into ROS that provides a safe and controlled environment for accurate modeling of real-world scenarios. The Cranfield University hangar environment was replicated in the Gazebo simulation for this experiment. The experiment for this project was conducted on a computer equipped with an Ubuntu 20.04 64-bit operating system with ROS noetic version, powered by an Intel(R) Core (TM) i7-10750H CPU at 2.60 GHz. For deep learning integration, the YOLOv7 tiny object detection model was used, and the RRT global path planning algorithm was also integrated, each at different interfacing nodes within the ROS costmap package. The setup utilized the Turtlebot3 waffle_pi robot package, which comes equipped with embedded LiDAR and camera sensors. For system validation, three sets of experiments were conducted to compare their performance and adaptability in an increasingly complex environment. The first approach evaluated the obstacle avoidance and path planning performance of the default ROS navigation stack comprising Dijkstra and DWA planner. The second approach combined the YOLOv7 object detection model with default ROS global and local planners (NAV-YOLO). The last method integrated the YOLOv7 model and RRT global planner into the ROS navigation stack (NAV-YOLO-RRT). Figure 4 shows an overview of the three solutions compared.

During the baseline experiment using the default ROS navigation stack, the robot’s movement and interactions with obstacles, as well as sensor data, were systematically recorded with conditions that are consistent with other experiments, including starting point and goal location. The NAV-YOLO and NAV-YOLO-RRT experiments followed the same procedure but involved an object detection model that generates transformed laser scan data from the RGB-D images used for the evaluation of obstacle detection performance. In the experiment environment, five distinct checkpoints were defined, representing inspection points around the aircraft. The environment is generally populated with 20 different and strategically positioned obstacles comprising 10 statics and 10 dynamic obstacles. These obstacles were incrementally added, creating three different environments to assess the robot’s behavior in environments of increasing complexity. The three environments (1, 2, and 3) were built with ten static obstacles each and three, six, and ten dynamic obstacles, respectively. The experimental setup is structured to guide the robot through predefined checkpoints and then to the target location, which is also the starting point. This is to evaluate the robot’s path planning and obstacle avoidance capability through the generated route. The use of “rosbag” plays a crucial role in this process and was used to capture relevant data that was used for the evaluation. This includes measuring metrics like the time taken to reach the target location, distance covered, and collision rate, which are necessary for assessing the efficiency of the robot’s navigation system in real-time hangar scenarios.

### 4.1. Data Collection

The data were collected to comprehensively analyze the robot’s behavior, navigation performance, and the accuracy of the object detection system. The robot’s Adaptive Monte Carlo Localization (AMCL) pose was recorded using the ‘rosbag’ and converted to a CSV file to determine the robot’s position and orientation in the environment. AMCL is a robotic algorithm that uses a particle filter to estimate a robot’s position in its environment [41]. In each environment, the ‘rosbag’ records key metrics, including sensor data, robot pose, goal status, and time to reach goals for effective performance evaluation. The AMCL pose of the robot was used to plot and compare the path taken for each of the compared methods. The total distance over time was computed using the Euclidean distance formula shown in Equation (5). A motion script was developed that initiates a timer at the beginning of the navigation task to its destination to measure the total navigation time that is used to evaluate the fastest approach. Another script was also used to log each collision event, incrementing a counter every time a collision is detected. The data collection experiments of this project are structured to ensure that the results are consistent and reliable and are conducted multiple times under the same initial conditions. Upon completion of the experiments, the gathered data undergo rigorous analysis to derive meaningful conclusions.

### 4.2. Results

To assess the efficacy of the proposed solutions, comparative analyses were conducted, using the ROS navigation stack as the benchmark. The choice was a result of an established standard and framework for ROS navigation stack that is widely adopted. Leveraging it as a baseline will give researchers practical insight and understanding of the improvement and efficiency offered by the proposed solution. Three different simulated environments with a different number of obstacles were used. The evaluation was based on three critical metrics: the time to reach the target location, the total distance covered during navigation, and the number of collisions encountered. The start position of the robots was consistently set at the coordinates of (6.0, 7.0, 0.0) in each experiment, ensuring accurate comparison of the system’s performance across different environments, including Environments 1, 2, and 3 used in this project experiment. In Environments 1 and 2, which have ten static and three dynamic and ten static and six dynamic obstacles, respectively, the default ROS navigation stack encountered collisions at two different points and along with notable increase in time and distance covered. This signifies its limitation in obstacle detection, avoidance, and efficient path planning. Conversely, the other developed solutions guided the robot through the environments within reasonable timeframes and distances without colliding with obstacles. In Environment 3, the complexity increased with 10 static and 10 dynamic moving persons as obstacles. Both the ROS navigation stack and NAV-YOLO solutions encountered collisions in Environment 3, surpassing the collision occurrence observed in Environments 1 and 2. Furthermore, they require more time and distance to navigate to the target location as environmental complexity increases when compared to the navigation experience in the earlier environments.

**Figure 5:**
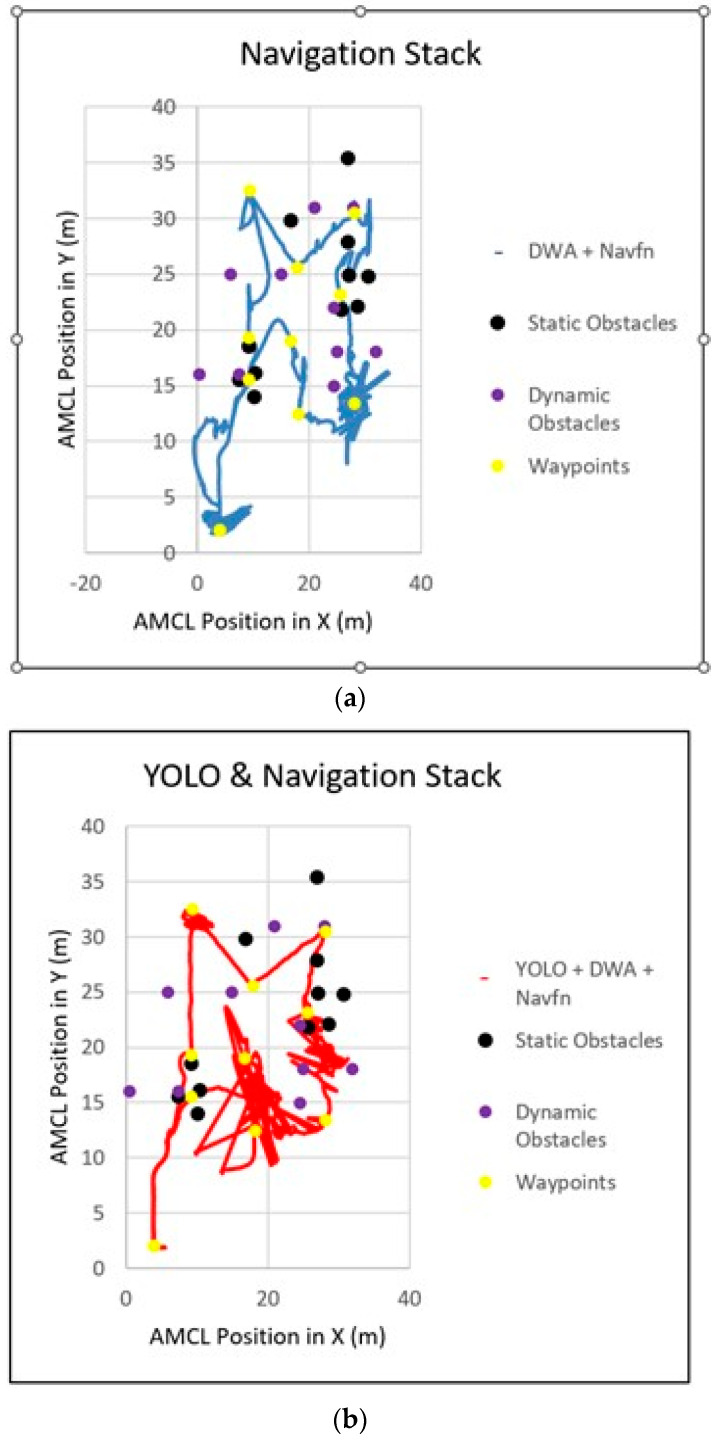

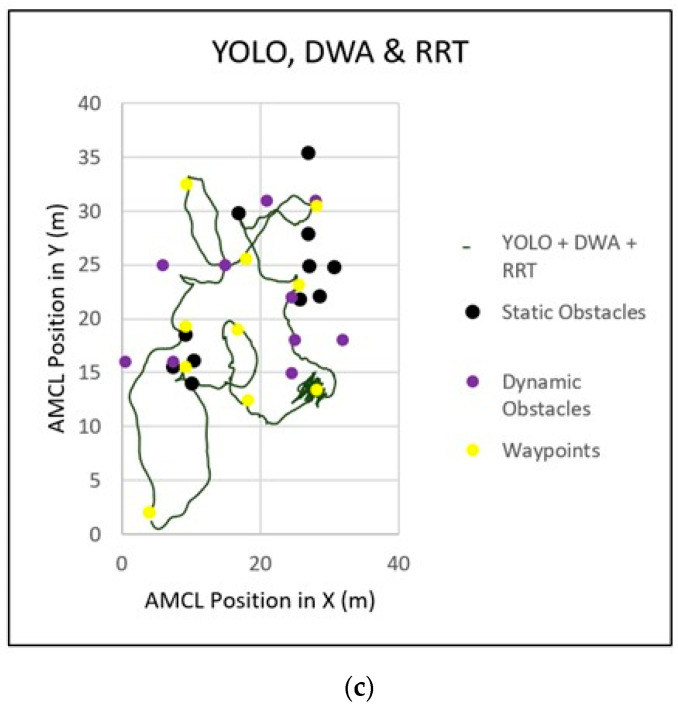
(**a**) ROS Navigation stack trajectory. (**b**) NAV-YOLO trajectory. (**c**) NAV-YOLO-RRT trajectory.

The proposed NAV-YOLO-RRT solution effectively navigated to the target location with no collision encountered in Environment 3, as demonstrated in Table 1. Further comparisons in Figure 5a–c show the smooth trajectory generated and followed by the proposed solution, while the other solutions show very rough trajectories, indicating inconsistency in replanning routes and avoiding obstacles. This shows the efficiency of the NAV-YOLO-RRT solution in safely navigating complex environments with the improved obstacle avoidance mechanism. However, the proposed solution exhibited a longer time and distance covered, spanning 18 min and 627.8 m, respectively, in Environment 3 when compared with the default ROS navigation and NAV-YOLO solutions, which covered distances of 619.5 and 615.8 m, respectively, in less time. This result outcome suggests that while the proposed solution achieved collision-free navigation, the complexity of the environment influenced the path planning strategies and required more time to reroute to the target location. This can be improved to enhance the real-time capability of the system.

### 4.3. Obstacle Detection Performance

**Figure 6:**
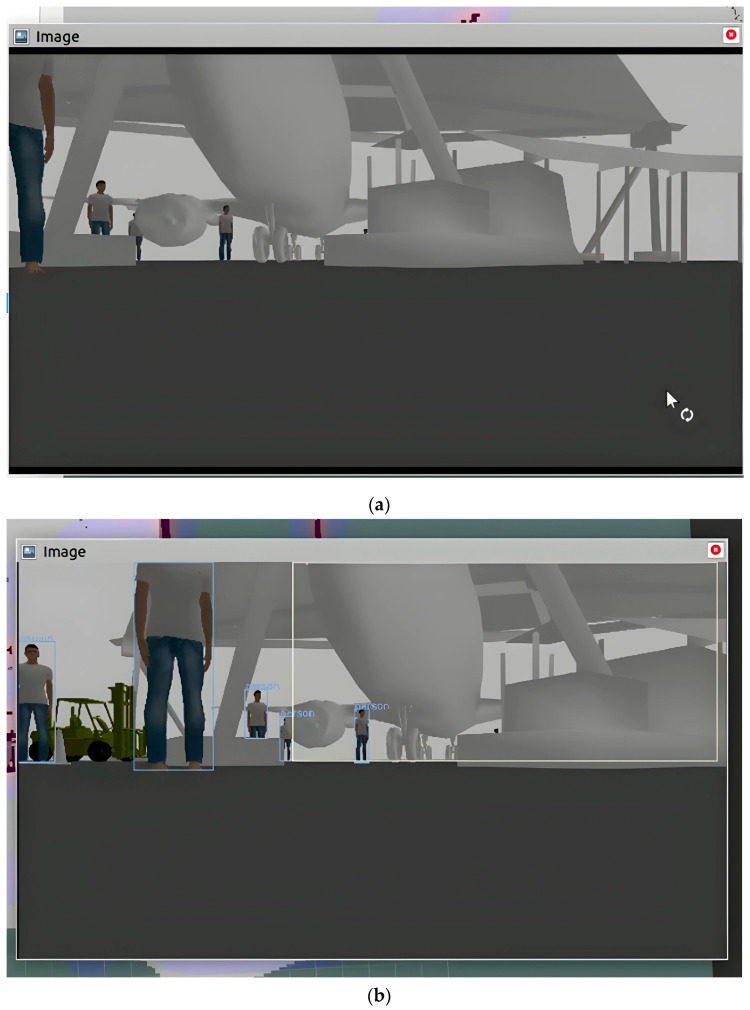
(**a**) Environment perception from camera view. (**b**) Environment object detection from YOLO.

**Figure 7:**
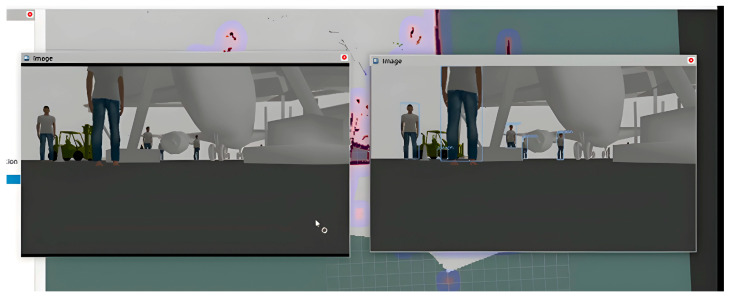
Improved obstacle detection with NAV-YOLO-RRT.

We further compared the obstacle detection and data synchronization capability of NAV-YOLO and NAV-YOLO-RRT, as shown in Figure 6 and Figure 7, to ensure a robust evaluation of the detection performance. For the reliability and validity of the experimental results, the experiments were conducted multiple times under the same initial conditions. This provides a robust understanding of any variation in the environment and the robot’s behaviour under different scenarios. We conducted the experiment integrating YOLOv3, YOLOv5, and YOLOv7 versions of the YOLO object detection model. The comparison between YOLOv3 and YOLOv5 in terms of inference time per frame highlights a significant difference in processing speed. YOLOv3 had an average inference time of 0.582 s per frame, while YOLOv5s demonstrated a faster average inference time of 0.225 s. Comparing the proposed NAV-YOLO-RRT solution integrated with YOLOv5 and YOLOv7, it was observed that the average time for the robot to reach its destination was approximately 17 min; during this period, the performance of the models in accurately identifying obstacles was closely monitored, including tracking metrics like frames processed per second (fps), correct detection, and incorrect detection, as shown in Table 2. YOLOv5 showed approximately 66% accurate detection with a faster inference rate of 168 on Turtlebot3, while in YOLOv7, a lower inference rate of 134 and approximately 70% improved accuracy were observed.

Further comparison between NAV-YOLO and NAV-YOLO-RRT showed that there were lags in obstacle detection in the robot’s view as it navigated the environment with the NAV-YOLO solution. Figure 6a shows the camera view, while Figure 6b shows the YOLO-detected obstacles in real time. The object views in the two images do not correspond due to synchronization issues between the sensor input and the transformed detected data. This implies that the processing of data from the point of the robot capturing images with the camera to YOLO processing, distance estimation, transformation from camera coordinates to laser scan data, and to navigation decisions posed some delays. For the NAV-YOLO experiment, hardware limitations, the YOLO model, and environmental complexity were significant contributing factors.

The experiment with NAV-YOLO-RRT, as demonstrated in Figure 7, showed improved data synchronization and object detection in real time. The environment perception from the camera view and YOLO object detection perspective tend to align reasonably in real time.

### 4.4. Path Planning Evaluation

The experiment also provided valuable insight into the robot’s path generation capability and trajectory, which were analyzed using the RVIZ visualization tool while moving through five predefined checkpoints toward its target location. For the evaluation, three distinct environments with increasing levels of complexity were created, ensuring a thorough assessment across various scenarios. Each experiment was repeated five times to minimize deviations and obtain a more accurate understanding of the system behavior at different instances. The key metrics used in this evaluation include average path length, time taken, and observed deviation from the planned trajectory. The comparative result illustrated in Table 1 showed three collisions with the default ROS navigation stack and two with NAV-YOLO in the most complex environment with dynamic and static obstacles. Although the NAV-YOLO system showed better performance in terms of completing the task in a shorter distance and time compared to other evaluated solutions, NAV-YOLO-RRT showed no collision. The proposed NAV-YOLO-RRT solution, on the other hand, displayed a slightly longer average completion time of 18 min compared to the NAV-YOLO and baseline systems, which averaged approximately 16 and 17 min, respectively. The obvious contributing factor was that the solution accumulated additional processing time while replanning the path to accurately avoid obstacles.

### 4.5. Transfer to Physical Turtlebot3 Waffle_pi

**Figure 8:**
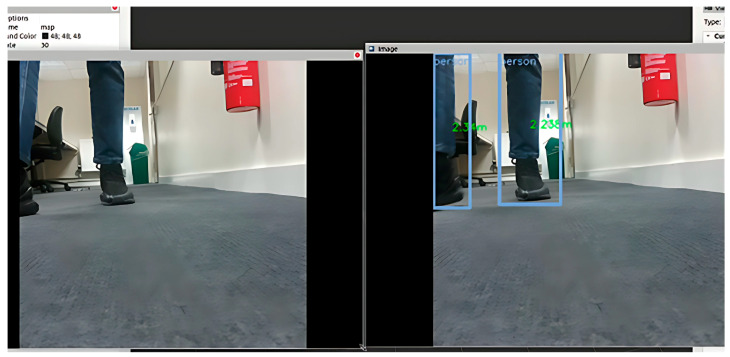
Detected and estimated obstacle positions.

**Figure 9:**
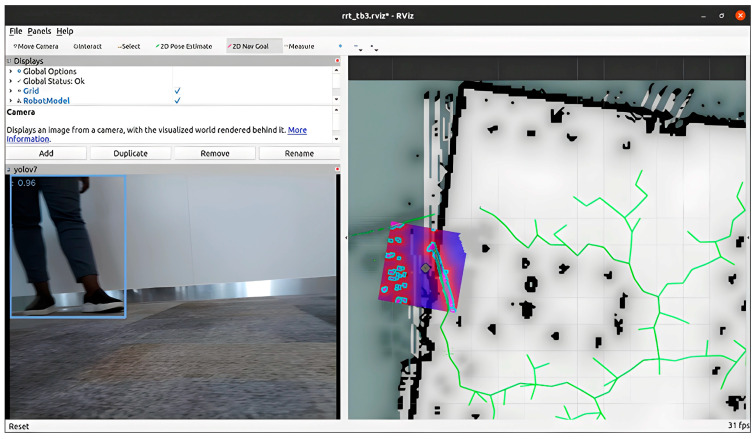
NAV-YOLO-RRT detection and path planning in the real-world robot.

The NAV-YOLO and NAV-YOLO- RRT solutions were transferred to the turtlebot3 robot at different instances for practical application and performance validation. The DepthAI Oak-D camera was used in the setup to enhance the robot’s vision capabilities. The NAV-YOLO-RRT demonstrated proficiency in detecting and estimating obstacle positions, aligning with the camera data stream, as shown in Figure 8. The system computes the pose of the detected obstacle within the laser scan frame by leveraging the bounding box center of the detected object, as illustrated in Equation (8). This helps the robot to navigate and avoid obstacles efficiently, even in unknown office environments, as shown in Figure 9.

## 5. Discussion

Analyzing the performance of the developed solutions relative to the baseline set by the default ROS navigation stack experiments offers valuable insight. This provides a comprehensive understanding of its effectiveness and the challenges faced in complex navigation scenarios. The proposed system operates at approximately 0.3 Hz frequency, which translates to about one frame every 3 to 4 s. This rate indicates the frequency at which the system responds to environmental data and is observed to achieve smooth navigation with less path replanning through to the target point. However, the 3- to 4-s delay introduced noticeable latency in its reaction, which could potentially impact obstacle detection and avoidance. This could be problematic considering the real-time responsiveness required in a dynamic hangar environment. In the next phase of the project, there will be a focus on utilizing improved prediction algorithms and an upgraded robot platform. From the integrated YOLO model’s perspective, there was observed disparity in inference time between YOLOv3 and YOLOv5, which can be attributed in part to the architectural improvement in YOLOv5 with CSP-Darknet53 as the backbone model [42,43]. YOLOv5 also demonstrated higher computational efficiency compared to YOLOv3, requiring 15.9GB@416 FLOPs (Floating Point Operations) against 154.9GB@416 at the same FLOP counts required.

While YOLOv5 offers a higher potential speed of 170 fps, suitable for real-time processing demands, YOLOv7, despite a lower frame rate of 144, provides better accuracy in obstacle detection and is more suited for the resources-constrained Turtlebot3 robot platform due to its lower computational requirements. However, there would be a need for trade-offs and potential optimization for the integrated NAV-YOLO-RRT system to be more efficient in the hangar environment. A focus will be on improving its average completion time and distance covered compared to other solutions. While the system offers superior obstacle avoidance ability that reduces the risk of collisions, enhancing safety and adaptability is important for real-world hangar applications, and improving navigation speed will be beneficial.

## 6. Conclusions

In this paper, the YOLO model was trained with custom datasets and integrated into the ROS framework to develop an improved obstacle detection, avoidance, and path planning system tailored for mobile robots in complex and dynamic hangar environments. This is to address the issue of the increasing complexity of real-world busy hangar environments, where detecting and avoiding obstacles of diverse structures and dynamics is quite challenging. Firstly, we replaced the customized YOLOv5 in an established NAV-YOLO solution with a customized YOLOv7 object detection model to improve the obstacle detection capability of the default ROS navigation stack. Second, fused LIDAR input with the transformed YOLO-detected objects to expand the robot’s field of view. Then, the proposed NAV-YOLO-RRT was developed, effectively leveraging the RRT planner to adapt to varying environmental conditions and dynamically replan trajectories to update the navigation path based on real-time sensor input. The experimental results demonstrated that the integration of the YOLOv7 with the mobile robot navigation system significantly improved the robot’s ability to accurately detect and avoid obstacles. The solution can enable the robot to respond promptly to changes in the environment, minimizing the risk of collision and improving overall navigation performance in the changing and dynamic hangar environments.

The mobile robot can autonomously navigate through predefined checkpoints, detecting and avoiding static and dynamic obstacles accurately, generating a safe path to the target location. This achievement marks a significant contribution to the field of robotics, particularly in enhancing autonomous navigation capabilities in challenging scenarios. In future work, the focus will be on improving the NAV-YOLO-RRT navigation speed, considering a balance with detection accuracy to enhance safety as well as operation efficiency in the hangar environment. Also, addressing latency in data processing, which impacts the robot’s ability to replan paths promptly in response to sudden obstacles, is another critical area. In addition, sensor fusion extension and hardware consideration will be part of the focus areas for future work.

## Figures and Tables

**Table 1:** Performance evaluation of developed solutions with baseline.

	Time (min)	Distance Covered (m)	Number of Collisions
ROS Navigation stack	17.57	619.5	3
NAV-YOLO	16.37	615.8	2
NAV-YOLO-RRT	18.0	627.8	0

**Table 2:** Performance results of YOLOv7 and YOLOv5.

Models	Frame Rate	Accurate Detection	Incorrect Detection
YOLOv5s	168	15	5
YOLOv7-tiny	134	17	3

## Data Availability

Data are contained within the article.
